# Planarian brain regeneration as a model system for developmental neurotoxicology

**DOI:** 10.1002/reg2.52

**Published:** 2016-03-15

**Authors:** Danielle Hagstrom, Olivier Cochet‐Escartin, Eva‐Maria S. Collins

**Affiliations:** ^1^ Division of Biological SciencesUniversity of CaliforniaSan Diego, La JollaCalifornia92093USA; ^2^ Department of PhysicsUniversity of CaliforniaSan Diego, La JollaCalifornia92093USA

**Keywords:** Behavior, neurodevelopment, planarians, regeneration, toxicology

## Abstract

Freshwater planarians, famous for their regenerative prowess, have long been recognized as a valuable in vivo animal model to study the effects of chemical exposure. In this review, we summarize the current techniques and tools used in the literature to assess toxicity in the planarian system. We focus on the planarian's particular amenability for neurotoxicology and neuroregeneration studies, owing to the planarian's unique ability to regenerate a centralized nervous system. Zooming in from the organismal to the molecular level, we show that planarians offer a repertoire of morphological and behavioral readouts while also being amenable to mechanistic studies of compound toxicity. Finally, we discuss the open challenges and opportunities for planarian brain regeneration to become an important model system for modern toxicology.

## Introduction

Toxicological studies, particularly those focused on neurotoxicology, have predominantly relied on the use of in vivo animal models to assess potential adverse effects on human health. Traditionally, toxicological screens have been performed in rodents and higher mammalian models because of their evolutionary proximity to humans. However, because toxicity testing in these animals is ethically questionable, time‐consuming and expensive, it is impossible to use this approach to achieve the necessary coverage of the increasingly vast number of environmental toxicants. The non‐confidential portion of the Environmental Protection Agency's Toxic Substances Control Act Chemical Substance Inventory (http://www2.epa.gov/tsca‐inventory/) currently lists more than 67,000 chemicals that are manufactured or processed in the USA. Since it is projected that global chemical production will double within the next 24 years (Wilson & Schwarzman 2009) and new compounds will be continually added to the market, traditional toxicology testing is inadequate and new methodologies and systems are necessary to meet the demand.

To this end, in 2008 the Tox21 initiative (http://epa.gov/ncct/Tox21/) was launched. This multi‐government agency collaboration has two aims. First, it strives to more quickly and effectively characterize the molecular and cellular pathways involved in the toxicity of known compounds. Second, it fosters the development of reliable high‐throughput screening (HTS) assays to evaluate chemicals for which little or no testing has been carried out in the past. To achieve the desired coverage and mechanistic insight, both HTS in vitro assays as well as medium‐throughput screening (MTS) in alternative animal models are necessary. Alternative animal models, including invertebrates and lower vertebrates, are ideal for MTS as they are free of ethical dilemma, inexpensive to maintain and amenable to automation, leading to increased screening throughput at reduced cost. Importantly, since many genes and core pathways are conserved between these simpler organisms and humans, their amenability for molecular studies allows for mechanistic insight into compound toxicity (Lein et al. 2005).

Freshwater planarians have arisen as one such possible alternative animal model (Hagstrom et al. 2015). Planarians have fascinated researchers for centuries for their regenerative prowess and ability to reproduce asexually via binary fission (Newmark & Sánchez Alvarado 2002). Studies in the early 1900s used physical and chemical manipulations to gain insight into the animals’ physiological and regenerative properties (Child 1909,1911). These efforts led to the discovery of many of the different morphological and behavioral readouts used in planarian toxicology today.

Because planarians were found to be highly susceptible to substances added to their aquatic environment, this feature is frequently employed to monitor water quality in environmental studies (Kapu & Schaeffer 1991; Rivera & Perich 1994). After exposure to water samples, different markers at the molecular (Prá et al. 2005) or organismal levels (Knakievicz 2014) can be used to assess the water pollution level and identify specific pollutants.

In addition, it has been recognized for over 30 years (Best & Morita 1982) that planarians are well suited for studying the effect of chemicals on brain development and function. Planarians are one of the simplest organisms that display cephalization and are unique in their ability to regenerate their entire central nervous system (CNS) following tissue loss, damage or asexual reproduction (Cebrià 2007). For asexual planarians, regeneration is the sole mechanism of neurodevelopment. This complex process of de novo neuroregeneration involves many of the same processes that occur during vertebrate neurodevelopment: stem cell migration, proliferation and differentiation and axonal guidance (Cebrià & Newmark 2005; Cebrià 2007; Umesono et al. 2011). Thus, neurodevelopment can be “induced at will” by amputation allowing toxicological studies to be performed directly on free‐living developing animals, without the complications of maternal effects.

Importantly, planarians provide a variety of quantifiable morphological, behavioral and molecular endpoints to analyze toxic effects on different aspects of development. Furthermore, the planarian CNS shares many of the same neurotransmitters and neuronal populations with the mammalian brain (Cebrià et al. 2002; Mineta et al. 2003; Cebrià 2007). For example, it has been demonstrated that neuromuscular communication is under the control of acetylcholine in planarians as in humans (Nishimura et al. 2010). Thus, mechanistic studies in planarians can provide insight into relevant mechanisms in humans.

Together, these characteristics render planarians specifically valuable as a model for developmental neurotoxicology. Importantly, because amputated and intact worms are of similar size, behavioral assays can be performed in parallel on both adult and regenerating/developing animals to determine whether chemicals, or particular concentrations, are specifically toxic or show greater potency to the developing brain. Because of this unique feature and its intermediate neuronal complexity, the planarian system is an ideal complement to existing alternative animal models in toxicology, such as zebrafish and nematodes, for which more extensive molecular toolkits are available (Hagstrom et al. 2015).

Complementary toxicology studies across multiple animal species are necessary to assay compound toxicity for humans, because species‐specific differences in sensitivity to toxicants exist (Hagstrom et al. 2015). High‐throughput, low‐cost alternative animal systems such as zebrafish, nematodes and planarians offer the opportunity to rapidly screen hundreds to thousands of potential toxicants to identify and prioritize candidates and mechanisms for further in‐depth studies in mammalian systems to assay their relevancy to humans (Lein et al. 2005). This approach will greatly enhance screening efficacy and thus save time and resources.

The goal of this review is to summarize the literature on toxicological studies in planarians, focusing on neurotoxicology. Zooming in from the organismal to the molecular level, we highlight behavioral, morphological, cellular and molecular readouts that have been used for the assessment of (neuro)toxicity. We end with a critical outlook on the limitations, challenges and opportunities of the planarian system for modern high‐throughput toxicology screens.

## Organismal readouts to study neurotoxicity in planarians

Signs of toxic effects are most readily observed at the whole organism level. Since planarians can be studied with the naked eye, organismal observations are accessible to manual scoring and have been used as early as the seminal studies by Child (Child 1909, 1911). Lethality is the most dramatic effect, but it is also the least informative readout about a compound's toxicity, because dead planarians generally disintegrate, hindering further examination. More interesting readouts include morphological aberrations, body distortions or changes in behavior, which may be specific to certain chemical classes (Passarelli et al. 1999; Raffa & Valdez 2001).

It is worth noting that body shape and behavioral readouts in planarians have also been used in pharmacology for decades (Raffa & Rawls 2008). In particular, locomotor activities have been extensively used by the Raffa laboratory to study the effect of numerous neuroactive drugs (caffeine, cocaine, etc.) on the planarian brain (Raffa et al. 2001; Pagán et al. 2012) as well as planarian withdrawal behaviors (Raffa & Valdez 2001; Raffa et al. 2003; Raffa & Desai 2005). For the purpose of this review, we will focus solely on the field of toxicology and we refer the interested reader to the reviews by Buttarelli et al. (2008) and Raffa and Rawls (2008) for details on pharmacological studies in planarians.

**Figure 1:**
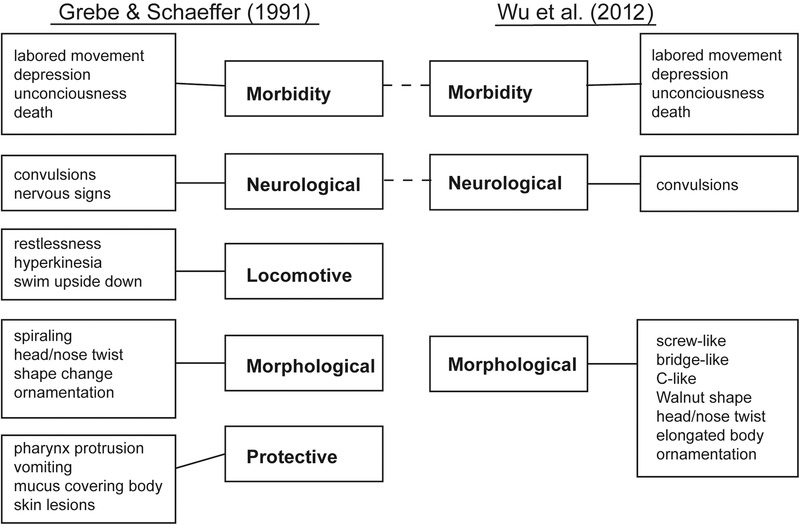
Comparison of the two scoring systems by Grebe and Schaeffer (GS system) (Grebe & Schaeffer 1991) and Wu et al (2012a). While substantial overlap exists between the two systems, the GS system provides more readouts (18 vs. 13). The Wu system has the advantage of clear categories of shape changes.

In 1991, Grebe and Schaeffer (1991) introduced the first qualitative scoring system to assess organismal toxicity of phenol in planarians. This landmark paper provided a matrix for analyzing compound toxicity using well‐defined criteria, which has since been used by a large number of researchers (Kapu & Schaeffer 1991; Villar et al. 1993; Pagán et al. 2006). The Grebe−Schaeffer (GS) scoring system comprises five main categories: locomotive, morphological, neurological, morbidity and protective (Fig. 1). Each of these categories contains between two and five criteria to be assessed visually, providing 18 different readouts to describe compound toxicity. Combinations of some of these readouts had been used previously in planarian toxicology by numerous groups; however, the GS system was the sole scoring system incorporating all of them.

**Figure 2:**
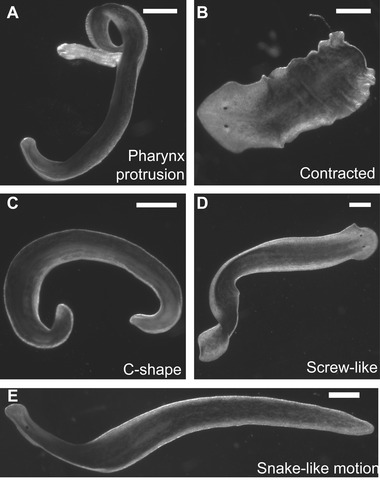
Examples of planarian morphological readouts and body shapes. (A) Pharynx is extended outside of the body. Animal treated with 0.4% chloretone. (B) Body is contracted. Often associated with wrinkles/ornamentation in the periphery of the animal. Animal treated with 20 μmol/L chlorpyrifos oxon. (C) Body is curled in a C‐shape. Also referred to as a banana curl or coil. Animal treated with 0.4% chloretone. (D) Body is twisted around itself in a screw‐like fashion. Also referred to as spiraling. Animal treated with 100 mmol/L serotonin. (E) Animal is extended and moves along its side in a snake‐like motion. Animal treated with 100 mmol/L serotonin. Scale bar 0.5 mm.

Most recently, Wu et al (2012a), studying cadmium toxicity in planarians, modified the GS scoring system by condensing most of the same readouts into three main categories (morphological, neurological, and morbidity). According to the Wu system, morphological readouts contain both body shapes and behavior, whereas neurological readouts only contain convulsions (Wu et al. 2012a). One of the advantages of the Wu system is the clear definition of possible shape changes (C‐like, screw‐like, etc.; see Fig. 2). However, the readouts of the “protective” category of the GS system were dropped. This is unfortunate, because more readouts improve the robustness of toxicant categorization.

Therefore, we prefer a classification system containing all GS system readouts, keeping the same level of detail but using the three category labels of the Wu system (morbidity, morphological and neurological function). We incorporate “vomiting,” also known as defecation in the planarian community, into the original GS morbidity category, and the other readouts of the GS protective category into the GS morphological category. The latter thus contains all phenotypes manifested in changes in worm shapes. Finally, our neurological function category refers to all worm behaviors whereby translational motion is observed, thus comprising the GS locomotive and GS neurological categories. Below, we discuss each of these three categories in detail.

### Morbidity

The first step in toxicity screening is to determine which concentrations are lethal. This is frequently achieved through range‐finding tests to determine which concentrations are non‐lethal or to determine the concentration at which 50% of the animals die (LC_50_). The GS system not only includes death as a readout, but also potential death indicators, such as unconsciousness or pharynx protrusion, to refine the observations and capture the dynamics of lethality for certain chemicals (Fig. 1). Of note, pharynx protrusion does not always imply that death with follow.

Death is easily scored in planarians since the worms disintegrate after dying (Buchanan 1935), allowing for high‐throughput lethality assays. Various methods have been employed in the literature, ranging from manual scoring (Grebe & Schaeffer 1991; Alonso & Camargo 2011) to bulk studies (Pagán et al. 2006, 2009) and automated screening (Hagstrom et al. 2015). For example, in Pagán et al. (2009), planarians were distributed in four separated quadrants in a Petri dish with each quadrant containing six to seven worms in a different chemical and/or a different concentration. The fraction of worms alive at time *t* was determined by counting the number of worms in the quadrant. The data can be fitted to a classic Hill equation (Hagstrom et al. 2015) to obtain the desired LC_50_. This method allows lethality to be assessed quickly using several time points, concentrations and a large number of worms in a single lethality assay.

Because some chemicals may preserve the worm tissue, preventing complete disintegration, the approach above has limited sensitivity compared to a scoring system that also includes death indicators, such as the GS system. The latter, however, are difficult to score in an automated fashion and largely depend on manual visual inspection of individual worms, limiting the throughput capacity.

### Morphological readouts

The combination of morphological and behavioral readouts into a single category, as first proposed by Wu et al. (2012a), makes sense in so far as the morphological readouts reported in the literature can largely be defined as behavioral. For instance, criteria such as “body elongation” or “nose twist” (Grebe & Schaeffer 1991; Wu et al. 2012a) are not morphological in the sense of developmental malformations but, instead, are a consequence of improper muscle control (Passarelli et al. 1999). In contrast, body shape changes such as lesions, pharynx extrusions or wrinkles/ornamentation (Fig. 2A, B) (Grebe & Schaeffer 1991; Wu et al. 2012a) are not necessarily indicative of changes on the neuronal level. Thus, morphological readouts are a mixed category in the sense that some morphological changes are the result of improper neuronal functions while others are not. However, because all reflect in body shape changes, we prefer to keep them in one category.

Morphological readouts have been used in a variety of contexts in the literature. The first naming convention for specific shapes was introduced in 1989 by the Palladini group. Working on the dopaminergic system in planarians, they standardized terms for common morphological observations, including C‐like shapes (Fig. 2C; Venturini et al. 1989), screw‐like hyperkinesia (Fig. 2D; Venturini et al. 1989) and snake‐like motion (Fig. 2E; Passarelli et al. 1999; Wu et al. 2012a). These specific shape changes are a consequence of impaired neuromuscular control as has been shown in Venturini et al. (1989) and Buttarelli et al. (2000).

Although most morphological analysis has been done by eye, shape changes can be quantified using automated shape analysis. Because the body shapes are not always as distinct as in the examples shown in Figure 2, machine learning algorithms (Jeanray et al. 2015) may be necessary to achieve a reliable automated categorization of body shapes, as for example used for *Caenorhabditis elegans* phenomics (Wählby et al. 2012).

Overall, changes in worm shape are common tools in assessing the toxicity of chemicals on planarians. However, their observation has been qualitative and relied on visual inspection of the worms, which is slow, prone to observer bias and leads to small numbers of samples. In addition, because research groups use different scoring systems, it is difficult to compare results between studies.

### Neurological (behavioral) readouts

**Figure 3:**
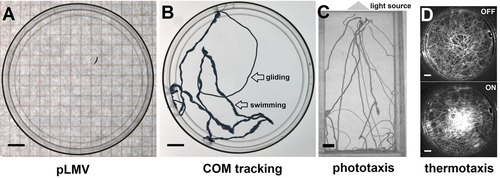
Overview of behavioral assays employed in the literature to quantify neuronal function after toxicant exposure. (A) The planarian locomotor velocity (pLMV) method measures worm speed by counting the number of gridlines crossed in a given time. (B) Center of mass (COM) tracking to determine type of locomotion, worm velocity and exploratory behavior. (C) Phototaxis is generally tested using a linear light gradient. (D) Thermotaxis can be tested using a Peltier element to generate a cooler center, which worms prefer. Scale bar 1 cm.

Unstimulated locomotion is probably the most accessible type of behavior in planarians. Without stimulation, planarians can rest, swim or glide (Hagstrom et al. 2015). These three behaviors can be distinguished by eye (Fig. 3B) and are informative about a chemical's effect on worm activity in general. Individual planarians, however, show intrinsically different preferences for resting, swimming and gliding under the same conditions (Hagstrom et al. 2015). Thus, unless a dramatic change in the relative frequency of these behaviors occurs or a significantly large sample size is studied, it is difficult to draw reliable conclusions regarding these behaviors. Similarly, a comparison of worm speed by the naked eye, as done in earlier studies (Child 1911; Grebe & Schaeffer 1991), is intrinsically subjective and unreliable when it comes to subtle changes in locomotion.

These limitations and the increased interest in planarians as a model for toxicology and pharmacology prompted the community to develop objective and quantitative measurements. The most extensively used technique to date is the planarian locomotor velocity (pLMV) method, first introduced by Raffa et al. (2001) and since used by a large number of research laboratories (Alonso & Camargo 2011; Zhang et al. 2013; Ramakrishnan et al. 2014; Lowe et al. 2015; Stevens et al. 2015). The pLMV method is similar to methods used for quantifying rodent behaviors; see for example Yamin et al. (2013). A single worm is placed in a standard 10 cm diameter Petri dish on top of a squared grid (primarily 0.5 cm or 1 cm wide mesh, Fig. 3A). The worm is allowed to move freely and the number of lines crossed in a given amount of time is recorded.

The same measurement can be performed on worms exposed to specific toxicants and directly compared to their wild type counterparts, providing a quantitative assessment of planarian activity. pLMV has been used to assess the toxicity of various chemicals, including ammonia (Alonso & Camargo 2011), dimethyl sulfoxide (DMSO) (Pagán et al. 2006; Stevens et al. 2015), cadmium (Wu et al. 2012a) and the dopamine D2‐receptor antagonist sulpiride (Raffa et al. 2001), as well as in the pharmacological study of the dopaminergic (Passarelli et al. 1999; Raffa et al. 2001), serotonergic (Farrell et al. 2008) and opioid systems (Buttarelli et al. 2002) in planarians.

The pLMV method allowed researchers to discover effects that would have eluded qualitative characterizations. However, it suffers from a few drawbacks. Counting line crossings that are 0.5 cm apart is imprecise for determining planarian speed. Also, across its many uses, the details of the pLMV technique have varied among groups through the use of different grid sizes and different time frames, making direct comparison of the results challenging. Moreover, pLMV only estimates absolute speeds but does not take into account the worm's trajectory, which could also yield important information, for example on the frequency of turning or exploratory behavior (Talbot & Schötz 2011). Finally, the scoring of line crossings is performed manually, leading to slow throughput.

In recent years, we (Talbot & Schötz 2011; Hagstrom et al. 2015) and others (Li 2012) have replaced pLMV with real‐time center of mass (COM) tracking, which allows the reconstruction of full worm trajectories to measure instantaneous velocities (Fig. 3B; Talbot & Schötz 2011; Hagstrom et al. 2015). This approach gives access to new properties of the worm's behavior such as the type of locomotion, for example swimming versus gliding (Fig. 3B and Hagstrom et al. 2015), the frequency of sharp turns and head wiggles or the time spent at the center versus the periphery of the testing arena (Talbot & Schötz 2011). It can thus reveal more subtle changes in behaviors and underlying defects in neuronal functions. Using COM tracking, we were able to show that two different compounds, sulpiride and chloretone, gave distinctively different phenotypes (Talbot & Schötz 2011). It is also worth noting that these last examples are quantitative measurements of readouts already present in the GS system (see nervous signs in Fig. 1).

Clearly, to assay the function of specific neuronal populations, studying gliding alone is insufficient. We have recently introduced a new planarian gait, scrunching (Cochet‐Escartin et al. 2015), which can be induced in a well‐controlled fashion using external noxious stimuli. Scrunching relies on coordinated muscular contractions and has a characteristic signature of asymmetric body contractions that can be quantified using simple image analysis tools. As such, it could become an important readout for both proper sensory apparatus and neuromuscular communication.

Furthermore, the Agata group has made significant advances in quantifying more complex behaviors in *Dugesia japonica*, including thermotaxis, chemotaxis, phototaxis and thigmotaxis (Inoue et al. 2004, 2015), and showed that these behaviors depend on neuronal activity (Inoue et al. 2015). Using binary combinations of the respective stimuli, they further showed that a hierarchy exists with chemotaxis as the predominant behavior (Inoue et al. 2015). We have applied similar semi‐automated assays to quantify phototaxis (Fig. 3C and Lambrus et al. 2015) and thermotaxis (Fig. 3D and Hagstrom et al. 2015) in *Schmidtea mediterranea* and *D. japonica*, respectively, and found that they are reliable readouts of neuronal function. Thus, even without any knowledge of the underlying toxicity mechanisms, behavioral assays allow assessment of whether proper neuronal functions are maintained after toxicant exposure.

Importantly, how these different behaviors are regulated at the neuronal level is beginning to be unraveled. For example, gliding was shown to depend on serotonin signaling (Currie & Pearson 2013), thermotaxis on TRPMa sensory neurons and serotonergic neurons (Inoue et al. 2014) and phototaxis on visual neurons and GABAergic neurons (Inoue et al. 2004). Because we have some insight into the neuronal control of these behaviors, it is imperative to incorporate as many behavioral endpoints as possible in the next generation of toxicology screenings to reveal dysfunctions of specific neuronal subpopulations.

Another future avenue is the use of conditioning in planarians. This field bloomed in the 1950s and 1960s with the seminal work of McConnell and coworkers (Thompson & McConnell 1955), who showed that planarians could learn simple tasks using classical conditioning (Thompson & McConnell 1955; Block & McConnell 1967). In addition, their experiments indicated that memories could be retained through brain regeneration (McConnell et al. 1959) and even be transferred across specimens through cannibalism (McConnell 1962). For a more detailed review, see Shomrat and Levin (2013). McConnell's studies, however, were executed manually and difficult to replicate and thus have remained controversial (Kartry et al. 1964; Walker & Milton 2013). However, a recent study by the Levin group, using automated tracking of a large number of worms, showed that planarians are capable of environmental familiarity. Furthermore, their results suggest that environmental familiarity may be sustained through brain regeneration (Shomrat & Levin 2013). Given the intrinsic variability in behaviors among even clonal planarians, more studies of this sort will be necessary to further explore learning and memory in planarians. The ability to test toxicological effects on such cognitive tasks would greatly broaden the scope of planarian toxicology.

## Neuroregeneration and neurotoxicology

Because of their unique neuroregenerative capabilities, and the large array of organismal readouts, planarians have prompted a growing number of toxicologists to study developmental neurotoxicity of natural and synthetic toxicants in this system over more than three decades (Best & Morita 1982; Schaeffer 1993). This has included studies of known or suggested developmental toxicants or teratogens such as ethanol (Hagstrom et al. 2015; Lowe et al. 2015), methylmercury (Best et al. 1981), *N*,*N*‐dimethlyformamide (DMF) (Zhang et al. 2013) and organophosphate pesticides (Villar et al. 1993; Hagstrom et al. 2015), known neurotoxicants such as DMSO (Hagstrom et al. 2015; Stevens et al. 2015), and substances with unclear toxicity such as the natural alkaloid berberine (Balestrini et al. 2014) or silver nanoparticles (Kustov et al. 2014). Although, at first, planarian toxicology relied primarily on qualitative characterization of gross morphological developmental defects (Best et al. 1981; Best & Morita 1982; Villar et al. 1993), more recent work has begun to address developmental toxicity through quantitative and mechanistic approaches.

**Figure 4:**
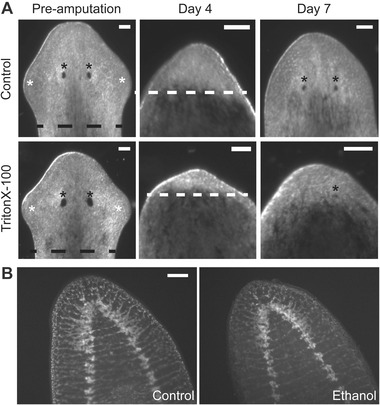
Morphological and anatomical readouts of developmental neurotoxicity in planarians. (A) Time course of regeneration of control (top) and 15 mg/mL Triton X‐100 treated (bottom) worms. On day 1, animals are amputated along the black dotted line. On day 4, the unpigmented blastema (indicated by the white dotted line) is clearly distinguishable. On later days, the reappearance of eyes (black asterisk) and auricles (white asterisk) can be scored. (B) Brain structure is visualized by immunohistochemistry with anti‐synapsin antibody (anti‐ SYNORF1, Developmental Studies Hybridoma Bank) in control and 0.1% ethanol treated regenerating animals 2 weeks post‐amputation. Scale bar 0.1 mm.

The oldest and simplest morphological characterization of regeneration has been used for decades in planarian toxicology studies (Villar et al. 1993). It was originally introduced by Child (1911) and consists of scoring the reappearance of head structures such as the eye spots or auricles, which typically reappear within 4−5 and 9 days of regeneration, respectively, in untreated animals (Inoue et al. 2004; Zhang et al. 2013) (Fig. 4A). Comparison of different morphological endpoints provides increased sensitivity as certain chemicals may affect the regeneration of one structure but not the other, as evidenced by the effect of DMF on auricle but not eye regeneration (Zhang et al. 2013). Auricles, however, are not equally striking in all planarian species, with *Dugesia dorotocephala* and *Dugesia tigrina* being the most apparent and *S. mediterranea* the least (Carter et al. 2015), limiting the applicability of this particular readout.

With the availability of higher resolution imaging techniques, quantitative morphological analysis has become possible. We and others have used high‐resolution light microscopy to quantify the rate of blastema growth during regeneration (Balestrini et al. 2014; Kustov et al. 2014; Hagstrom et al. 2015). Because the blastema is unpigmented, it can easily be distinguished from the rest of the pigmented worm body, allowing for automated image analysis (Balestrini et al. 2014; Kustov et al. 2014; Hagstrom et al. 2015). However, since different sized worms may have different regeneration rates, the size of the blastema must be normalized by worm size. While other groups have normalized by the area of the worm (Balestrini et al. 2014; Kustov et al. 2014), we found that normalizing by the square of the worm's width was the most accurate way to account for size variation (Hagstrom et al. 2015). Blastema growth rate is best used as an indicator of general developmental toxicity (Hagstrom et al. 2015) since it is not specific to neurodevelopmental defects per se. For example, we found that while a neurotoxic pesticide, permethrin, did not affect blastema growth rate, it did delay eye reappearance (Hagstrom et al. 2015).

In addition, developmental neurotoxicity can be characterized based on the return of different behaviors. As neuronal subpopulations are regenerated, specific behavioral functions are restored, allowing researchers to differentiate between the effects of different chemicals on neuronal subpopulations. This unique opportunity provided by the planarian system was recognized as early as 1982 by Best and Morita (Best & Morita 1982) and has since been utilized to study the effects of several neurotoxicants, including ethanol and DMSO (Hagstrom et al. 2015; Lowe et al. 2015; Stevens et al. 2015). However, as with studies on adult worms, these behavioral tests have been limited in their throughput and range of behaviors tested, primarily relying on pLMV and phototaxis (Balestrini et al. 2014; Lowe et al. 2015; Stevens et al. 2015). In a recent paper, we have used automated COM tracking to measure gliding speed, locomotion type and thermotaxis (Hagstrom et al. 2015), as a first step to overcome this limitation.

Importantly, behavioral tests can be conducted in parallel on both regenerating and intact animals allowing determination of development‐specific toxicity. For example, this type of comparison has led us and others to demonstrate the increased sensitivity of regenerating planarians to DMSO and ethanol (Hagstrom et al. 2015; Lowe et al. 2015; Stevens et al. 2015). These studies also demonstrate the importance of using multiple time points as developmental toxicity could be manifested as either the complete loss of a behavior or just delayed reacquisition.

In summary, automated behavioral testing allows for time and cost efficient screening of potential impairment of neuronal function before investigating the underlying mechanisms. Because the specific neurotransmitters and pathways involved in some of these behaviors have been determined (Inoue et al. 2004; Umesono et al. 2011; Inoue et al. 2015), characterizing behavioral neurodevelopmental defects upon neurotoxicant exposure can serve as a starting point for in‐depth analysis of the responsible molecular mechanisms.

These mechanisms can begin to be delineated using molecular localization techniques, such as in situ hybridization and immunohistochemistry, to characterize effects on specific developing anatomical and cellular structures. Pan‐neuronal markers such as synaptogamin and synapsin have been used to visualize gross toxic effects to the regenerating planarian brain (Balestrini et al. 2014; Hagstrom et al. 2015). Because of its structural simplicity, quantification of brain size can be used to quantify toxic effects on neuroregeneration (Balestrini et al. 2014; Hagstrom et al. 2015). However, this technique only provides information on gross anatomical defects, as we found with DMSO, permethrin, chlorpyrifos, ethanol, methanol, Triton X‐100 and acrylamide, but may not be sensitive enough to detect less obvious defects (Hagstrom et al. 2015). To address this issue, markers to specific neuronal subpopulations, such as the optic chiasm marker arrestin/VC‐1 (Agata et al. 1998) or the dopaminergic marker tyrosine hydroxylase, have been used to identify toxic effects specific to certain structures or neuronal subpopulations (Nishimura et al. 2011; Balestrini et al. 2014). The recent availability of a large array of planarian markers to specific neuronal populations (Cebrià et al. 2002; Robb & Sánchez Alvarado 2002; Ross et al. 2015) provides an exciting opportunity to perform more targeted mechanistic studies to analyze effects on specific neuronal subpopulations.

## Mechanisms and metabolism

One of the strengths of the planarian system is the ability to connect morphological and behavioral effects on the organismal level with effects on the molecular and cellular levels. Arguably, these mechanistic findings may be the most relevant aspect of planarian toxicology studies to human toxicology, particularly as core mechanisms are conserved.

Researchers have begun to investigate how various neurotoxicants affect important conserved molecular targets in planarians (Table 1). For example, several studies have analyzed how the activity of different biomarkers changes during the course of toxicant exposure (Yuan et al. 2012; Wu et al. 2012a; Zhang et al. 2014,2015) using colorimetric assays on homogenates of exposed animals. This technically simple approach has been used to assay important neurological enzymes (acetylcholinesterase and monoamine oxidase; Wu & Li 2015) and antioxidants involved in controlling oxidative stress (catalase, superoxide dismutase, glutathione peroxidase; Yuan et al. 2012; Zhang et al. 2014, 2015). Furthermore, Yuan and colleagues found that, while short exposures to moderate concentrations of DMSO increased antioxidant activity, longer exposures and higher concentrations significantly decreased activity (Yuan et al. 2012). These results emphasize the importance of testing several concentrations at different time points during exposure for a mechanistic understanding of toxicity.

**Table 1:** Mechanistic pathways tested in planarian toxicology

Pathways	Markers	Technique	Toxicants tested	References
Oxidative stress	Catalase (CAT), superoxide dismutase (SOD), glutathione peroxidase (GPX), glutathione (GSH), reactive oxygen species (ROS)	Colorimetric assays	Surfactants	Li 2008
			DMSO	Yuan et al. 2012
			Cadmium	Wu et al. 2012a
			Copper	Zhang et al. 2014
			1‐octyl‐3‐methylimidazolium bromide	Zhang et al. 2015
Lipid peroxidation	Malondialdehyde (MDA)	Colorimetric assays	Surfactants	Li 2008
			Cadmium	Wu et al. 2012a
			1‐octyl‐3‐methylimidazolium bromide	Zhang et al. 2015
Apoptosis	Caspase‐3	Colorimetric activity assays	Berberine	Balestrini et al. 2014
			Pain relievers	Wu & Li 2015
Nervous system	*Prohormone convertase 2 (pc2)*,	RT‐PCR, in situ	Berberine	Balestrini et al. 2014
	*synaptogamin* (*syt), glutamic acid decarboxylase (gad), retinal homeobox (rax), Orthopedia (otp),innexin‐3 (inx3)*	hybridization	DMSO	Stevens et al. 2015
Stem cell proliferation	Phospho‐histone H3, *pcna, innexin ‐11*	Immunohistochemistry,		
and maintenance	*(inx‐11), minichromosome maintenance‐2 (mcm2), bruno*	RT‐PCR	DMSO	Stevens et al. 2015
Cancer	*DNA mismatch repair (msh2), epidermal growth factor‐1 (egfr1), forkhead box O (foxo), nour‐darake (ndk)*	RT‐PCR	DMSO	Stevens et al. 2015

However, easily quantifiable biomarkers are not available for all toxicant‐affected pathways. Thus, expression‐based approaches, including in situ hybridization, quantitative reverse transcription polymerase chain reaction (RT‐PCR) and immunohistochemistry, have been used to characterize effects on a broader array of molecular pathways, such as cancer, neurodevelopment and stem cell maintenance (Stevens et al. 2015). These approaches allow for analysis of effects on a variety of cell and tissue types to narrow down precisely which populations are most affected and how. For example, using a combination of these approaches, Balestrini and colleagues reported that berberine toxicity may be a result of inhibition of metalloproteinases controlling extracellular matrix remodeling (Balestrini et al. 2014).

Expression‐based techniques in the literature consist of PCR‐based assays and structural localization studies. PCR‐based techniques such as quantitative RT‐PCR allow for rapid analysis of many different pathways in a short time, which is necessary for studies wherein the mechanisms of toxicity are completely unknown (Balestrini et al. 2014). However, since toxicity may manifest through anatomical malformations due to the functional inhibition of molecular targets that may be independent of changes to mRNA levels, localization studies are useful to identify structural and anatomical toxic effects.

Together, these techniques provide rapid insight into the mechanisms underlying the observed toxicity. By comparing morphological and behavioral readouts with biochemical and molecular readouts, we can begin to unravel how toxicants manifest their toxic effects.

To better extrapolate findings in planarians to understand how toxicants may affect human health, it must be determined whether planarians metabolize these xenobiotics similarly to humans. Planarians primarily absorb chemicals in the water by epithelial diffusion although chemicals can also be taken in by the pharynx (Kapu & Schaeffer 1991; Balestrini et al. 2014). Several studies have demonstrated that a variety of toxicants are indeed absorbed by planarians. However, studies thus far have primarily looked at toxicants which are easily detected, such as berberine which is naturally fluorescent (Balestrini et al. 2014) or heavy metals which can be detected by atomic absorption spectrophotometry (Wu et al. 2012a). The distribution and bioaccumulation of xenobiotics within the planarian body appear to be chemical‐specific, even among the same class, as cadmium was found to accumulate in the head while copper was evenly distributed throughout the planarian body (Wu et al. 2012a). As many chemicals are metabolically activated or converted after uptake, it remains to be determined whether planarians metabolize toxicants through similar mechanisms as humans. However, a few examples, particularly studies on the organophosphate chlorpyrifos (Hagstrom et al. 2015) and tobacco‐specific nitrosamines (Wu et al. 2012b), exist demonstrating that metabolism and/or activation of certain chemicals occurs in planarians similarly to in humans.

In humans, a large portion of xenobiotic metabolism is performed by cytochrome P450s (Raunio et al. 2015). Analysis of the *S. mediterranea* genome (Robb et al. 2008) shows that these enzymes are present in planarians, although it remains to be determined how similar these enzymes are to their human homologs.

In conclusion, planarians are a powerful system to investigate mechanisms of toxicity, particularly those specific to neurodevelopment. Importantly, the availability of both behavioral and molecular tools allows effects on the molecular and cellular levels to be linked to their functional effect on behavior.

## Challenges and opportunities: planarian neurotoxicology in the 21st century

While planarian toxicology has led to important insights and the development of tools with broad applicability for planarian research, it faces several limitations to meet the growing needs of modern neurotoxicology. In our view, at least three challenges need to be met if planarians are to play a significant role in the future: screening throughput and robustness, unification of methodology and mechanistic analysis.

### Challenge 1: Screening throughput and robustness

The main limitation to existing planarian toxicology studies is the lack of fully automated assays. Because most of the techniques employed rely on manual visual inspection of worms and are thus labor intensive, they have been largely applied to a single chemical (cadmium, phenol, DMF, DMSO, or ammonia) (Grebe & Schaeffer 1991; Alonso & Camargo 2011; Wu et al. 2012b; Zhang et al. 2013), the interaction of two chemicals (DMSO + toxicant [Stevens et al. 2015], caffeine + guarana [Moustakas et al. 2015]) or, rarely, to a single class of chemicals (surfactants [Li 2012], pain relievers [Wu & Li 2015]). As a result, our current understanding of the effect of environmental toxicants on planarians is very limited. Recently, we have analyzed nine known neurotoxicants (Hagstrom et al. 2015), spanning from pesticides to surfactants and alcohols, which to our knowledge is the broadest quantitative toxicology study performed in planarians to date.

To achieve the necessary throughput, full automation of experimental assays and data analysis, with minimal human intervention, are indispensable. To achieve robustness, two conditions need to be met: (1) the number of endpoints must be large enough to enable distinction between classes of neurotoxicants and (2) there must be enough replicates to eliminate false positives and experimental artefacts (Hsieh et al. 2015). The need for replicates will be easily met once automation is realized. To achieve the required repertoire of screening endpoints, we must both automate existing manual readouts and look for novel readouts that are accessible to quantification and automation. To date, the planarian community collectively has built up an array of valuable endpoints, including both morphological (e.g., pharynx extrusion, C‐shape, hyperkinesia, eye defects) as well as behavioral (e.g., thermotaxis, phototaxis, chemotaxis, scrunching, environmental familiarity) readouts that are amenable to automated quantification via image analysis. The execution of the experiments leading to these measurements, however, remains largely manual or semi‐automated. Therefore, a major effort will be required to find engineering solutions to integrate these assays into a fully automated screening platform. Not all assays will be amenable to such an automated approach. It is therefore important to determine which “array of assays” will provide the necessary coverage of readouts and can be standardized across research groups through automated solutions or agreed‐upon protocols for semi‐automated setups.

### Challenge 2: Unification of methodology

It is challenging, if not impossible, to compare results on the same neurotoxicant from existing studies, because various research groups use different planarian species most commonly *D. dorotocephala* (Best et al. 1981; Kapu & Schaeffer 1991; Villar et al. 1993), *D. tigrina* (Knakievicz & Ferreira 2008; Ramakrishnan et al. 2014; Moustakas et al. 2015), *S. mediterranea* (Plusquin et al. 2012; Lowe et al. 2015; Stevens et al. 2015) and *D. japonica* (Li 2012; Yuan et al. 2012; Zhang et al. 2013; Hagstrom et al. 2015) and different species may have different susceptibilities and behavioral responses (Rivera & Perich 1994). There also exists a lack of uniformity in the field with research groups applying different methods and using only some of the described readouts of the GS and Wu scoring systems, particularly lethality and overall activity (measured by pLMV) (Pagán et al. 2006, 2009; Alonso & Camargo 2011). Additionally, researchers have assessed varying durations of exposure, spanning from several minutes (Pagán et al. 2006) to over a month (Alonso & Camargo 2011). As we have shown (Hagstrom et al. 2015), acute and chronic compound toxicity can differ, but it is difficult to predict these differences a priori. This heterogeneity makes direct comparisons of results on the same toxicants basically impossible. Thus, one of the major challenges for the future is to standardize a battery of tests and one or two species for conducting toxicology studies. The zebrafish community faces similar difficulties, whereby laboratories have developed independent screening criteria and methodologies and the exact experimental details are often not reported (Padilla et al. 2011). Since, for planarian toxicology, tool development is an ongoing effort, we have the possibility to streamline procedures at an early stage within the planarian community.

### Challenge 3: Mechanistic analysis

While HTS of compounds for toxicological profiling is valuable by itself, an animal model greatly gains in value if it can also shed light on the molecular mechanism underlying a compound's neurotoxicity. In principle, the planarian system promises to allow for such mechanistic insight because the planarian CNS remains tractable on the cellular level and molecular pathways are likely to be simpler than in higher vertebrates. Very few examples exist (Yuan et al. 2012; Balestrini et al. 2014; Stevens et al. 2015), however, which link the phenotypic readouts of toxicants to an underlying molecular mechanism. Although several of the core pathways commonly affected by toxicants are conserved in planarians, more studies need to be conducted investigating whether toxicants’ targets and metabolism in planarians are comparable to those affected in mammals. This would be most easily achieved by studying well‐characterized toxicants, such as the ToxCast Phase I chemicals (http://www.epa.gov/chemical‐research/toxicity‐forecasting), to determine if developmental neurotoxicity in planarians correlates with developmental neurotoxicity in humans and other mammals and occurs through similar mechanisms. Such studies would provide a framework to classify chemicals with unknown toxicity and validate the relevancy of toxicology screens in planarians to provide a first indication of potential toxicity in humans.

In addition, modern technologies such as RNA‐seq, which is already applied to the planarian system for stem cell studies (Scimone et al. 2014; van Wolfswinkel et al. 2014), need to be tested in the context of toxicological screening to assay their value for identifying molecular targets. Currently applied techniques for studying the molecular mechanisms underlying toxicity, such as in situ hybridization and immunohistochemistry, albeit necessary for gaining insight into possible anatomical changes to the nervous system, are not amenable to HTS. In the interim, quantitative RT‐PCR is being used by some as an intermediate throughput solution (Balestrini et al. 2014; Stevens et al. 2015).

In summary, if we are able to meet these three challenges, even partially, exciting pay‐offs and opportunities await, some of which go well beyond the field of neurotoxicology. For example, HTS behavioral screening will be an important and indispensable tool for planarian pharmacology. Finally, robust behavioral readouts are necessary to characterize RNAi phenotypes. To date, most of these studies have remained qualitative. Having access to a battery of fast and reliable quantitative tests to assert the animal's behavior following gene knockdown will accelerate and improve accuracy in phenotype descriptions and shed new light on planarian biology.

The future of planarian neurotoxicology is bright, but there is a lot of work to be done if we are up to the challenge.
